# Recurrence of chronic encapsulated hematoma following cyst formation after stereotactic radiosurgery for brain arteriovenous malformations: a case report

**DOI:** 10.1186/s41016-025-00387-6

**Published:** 2025-01-13

**Authors:** Iñigo L. Sistiaga, Gregorio Catalán-Uribarrena, Silvia Gamba, Alejandro Carrasco, Laura Zaldumbide, Lorena Mosteiro, Iñigo Pomposo

**Affiliations:** 1 Department of Neurosurgery, University Hospital Cruces, Bilbao, Basque Country, Spain; 2 Biocruces Bizkaia Health Research Institute, Bilbao, Basque Country, Spain; 3 Department of Surgery, Radiology and Physical Medicine, University of The Basque Country, Leioa, Basque Country, Spain; 4 Department of Pathology, University Hospital Cruces, Bilbao, Basque Country, Spain

**Keywords:** Cyst formation, Chronic encapsulated expanding hematoma, Stereotactic radiosurgery, Arteriovenous malformation, Late complication

## Abstract

**Background:**

Delayed radiation-induced complications after stereotactic radiosurgery (SRS) for arteriovenous malformations (AVM) have scarcely been described in the literature, and their incidence, pathophysiology, and treatment remain unclear. Additionally, the literature regarding these complications is confusing. The authors present a well-documented case report describing these late complications, adding evidence to the possible common pathophysiological mechanism underlying them, and illustrating an effective treatment modality when they occur.

**Case presentation:**

A case of a 28-year-old male with an increasing cyst formation (CF) appearing 10 years after SRS for AVM is presented. Despite surgical treatment, due to the incomplete resection of the angiomatous nodule, recurrence as a chronic encapsulated expanding hematoma (CEEH) occurred. This relapse required a second treatment, which could have been avoided if aggressive surgical treatment had been performed initially.

**Conclusions:**

This case highlights the continuum between CF and CEEH, challenging existing confusion in the literature. Complete resection of the angiomatous nodule associated with CF is imperative for achieving resolution and preventing recurrence.

## Background

Stereotactic radiosurgery (SRS) has become a well-established treatment modality for brain arteriovenous malformations (AVMs), especially for those located in eloquent areas [1]. While early complications have received significant attention, delayed radiation-induced complications such as late cyst formation (CF) and chronic encapsulated expanding hematomas (CEEH) following AVM SRS have been minimally explored in the literature. CF refers to the development of a fluid-filled cavity near the original AVM nidus after complete obliteration of the AVM [2]. CEEH, on the other hand, involves a subacute, expanding hematoma surrounded by a capsule, identified on MRI by low or mixed-signal intensity on T2-weighted images [3, 4]. Their incidence, pathophysiology, and optimal treatment strategies remain poorly understood [2, 3, 5–11]. Additionally, the literature regarding these complications is confusing. Despite their common features and clinical similarities, they have been described independently and the relationship between the two is uncertain [2, 3]. In spite of an initial asymptomatic presentation, given the potentially significant clinical repercussions and the eventual necessity of surgical treatment, these complications should be considered and suspected.

We here present a well-documented case describing the possible clinical and radiological presentation of a CEEH recurrence despite surgical treatment of CF secondary to AVM SRS. Furthermore, we illustrate an effective treatment modality when they occur, adding evidence to the possible common pathophysiological mechanism underlying these late complications.

## Case presentation

We present the case of a 28-year-old male who presented with an enlarging cyst formation during follow-up at our institution. In August 2009, he experienced a 3-day headache, prompting a computed tomographic (CT) scan that revealed a left occipital hematoma. Subsequent intra-arterial digital subtraction angiography (DSA) confirmed the presence of an occipital AVM. The patient underwent SRS using a linear accelerator (LINAC) at another institution, with a maximum dose of 16.5 Gy and a margin dose of 15 Gy. Despite this initial treatment, residual nidus was observed during follow-up, leading to a repeat LINAC SRS procedure in August 2013, with a maximum dose of 15.5 Gy and a margin dose of 15 Gy. Throughout this period, the patient remained asymptomatic.

Five years after the initial SRS treatment, in August 2014, the patient developed a three-day episode of right homonymous hemianopsia accompanied by a headache. Magnetic resonance imaging (MRI) revealed perilesional post-contrast enhancement and T2 hyperintensity, consistent with radiation-induced changes (RIC) and radionecrosis. Subsequent serial MRI follow-up demonstrated the stability of these findings and a control DSA confirmed the total exclusion of the AVM.

**Fig. 1:**
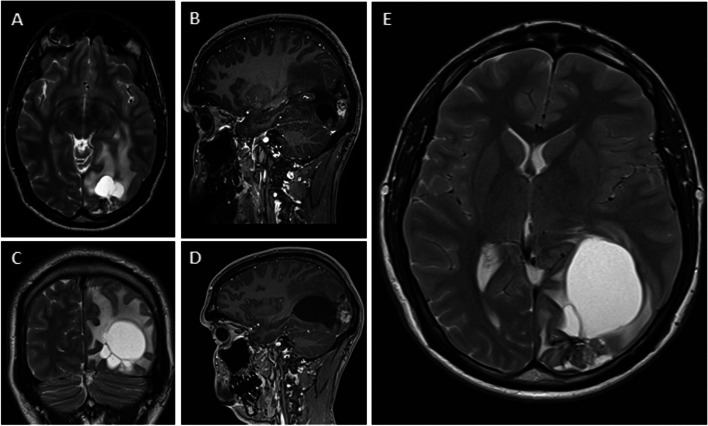
Serial MRI after AVM treatment with SRS showing an enlarging left occipital cystic formation with mass effect and edema. **A** Axial T2-weighted and **B** sagittal gadolinium-enhanced T1-weighted MRI 10 years after SRS treatment showed a cystic formation (11 × 16 × 15 mm) surrounded by vasogenic edema, adjacent to a contrast-enhancing angiomatous nodule. **C** Coronal T2-weighted, **D** sagittal gadolinium-enhanced T1-weighted, **E** axial T2-weighted MRI 1 year later (11 years post-SRS treatment) showing a significant enlargement of the cystic formation (70 × 35 × 50 mm), now multiloculated, and of the associated vasogenic edema with contralateral midline shift and incipient subfalcine herniation. The contrast-enhancing angiomatous nodule persists

However, in 2019 (10 years after the initial SRS treatment), serial MRI scans revealed a symptomatic, slowly-growing cyst formation adjacent to the radiated AVM, accompanied by significant mass effect and edema. Within the cyst, small pseudonodular T2-hyperintense formations were observed (Fig. 1). Despite treatment with dexamethasone, the patient continued to experience focal seizures affecting visual function.

**Fig. 2:**
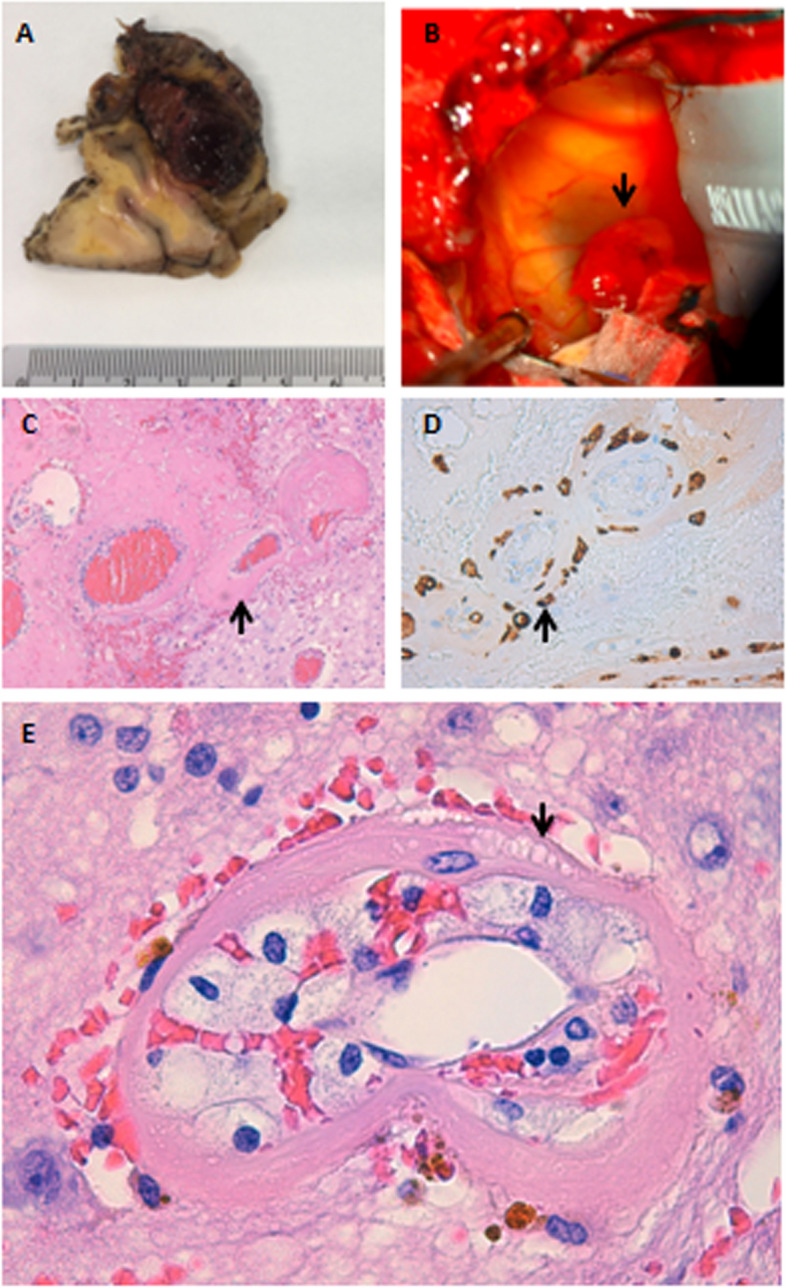
**A** Organized subacute hematoma embedded in the occipital lobe. **B** Intraoperative image showing the interior of the xanthochromic cyst and the angiomatous nodule (black arrow) corresponding to the contrast-enhancing area on MRI. **C**–**E** Histopathological images of hematoxylin–eosin staining showing dilated vessels with hyalinization of the vascular wall (**C** black arrow) with the presence of foamy histiocytes (macrophages) in the wall (**E** black arrow). **D** Immunohistochemistry showing histiocytes identified with CD-163

As a result, neurosurgical intervention was pursued in June 2020. Intraoperatively, the lesion appeared as a multiloculated cyst containing yellowish content suggestive of chronic bleeding. A nodular angiomatous lesion was evident (Fig. 2), corresponding to the area of contrast enhancement observed on MRI. The surgical procedure involved an uneventful craniotomy, subtotal resection, and fenestration of the multiloculated cyst. Due to the risk of permanent hemianopsia, a more aggressive approach involving total resection of the angiomatous nodule was avoided. Post-surgical assessment and follow-up MRI scans at 3 months and 6 months, revealed continued reduction of perilesional edema and shrinkage of the cystic component, with a remaining porencephalic area and partial persistence of the contrast-enhancing nodule. The patient improved clinically and was managed with a 2-week taper of dexamethasone alongside maintenance therapy with Keppra 500 mg BID. He was able to resume his job and showed good recovery.

**Fig. 3:**
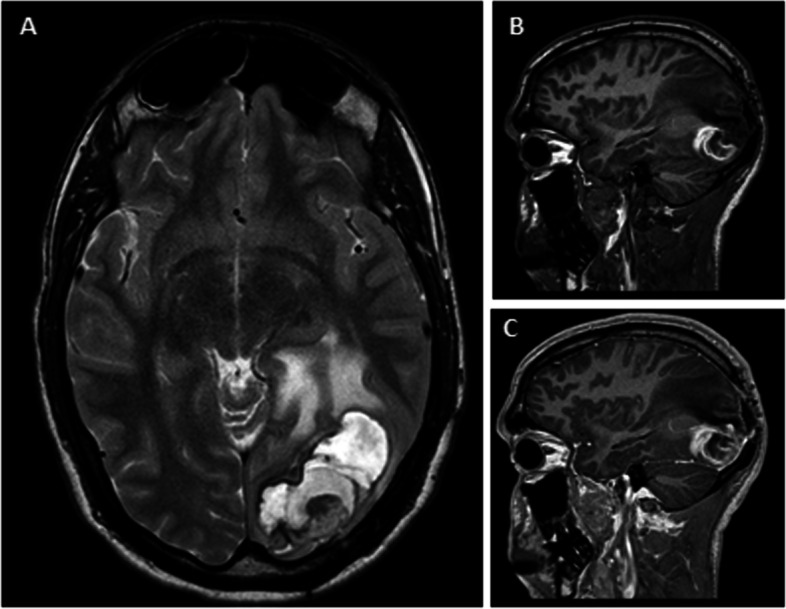
**A** Axial T2-weighted, **B** coronal T1-weighted, **C** coronal gadolinium-enhanced T1-weighted 1 year post-surgical MRI (12 years post-SRS treatment) showing a left occipital chronic encapsulated expanding hematoma (CEEH) with the persistence of the enhancing angiomatous nodule. Note the contrast-enhancing capsule

Unexpectedly, 1 year later (12 years post-SRS), the patient experienced an acute episode of intense, persistent headaches. Two months later, he developed a cluster of secondary generalized focal seizures. Repeat DSA confirmed complete occlusion of the AVM. Subsequent MRI revealed a subacute hematoma in the region adjacent to the contrast-enhancing nodule, accompanied by vasogenic edema (Fig. 3).

These findings align with previously reported radiological features of a CEEH. Due to the ongoing clinical persistence of seizures and progressive visual impairment, reintervention for the patient was deemed necessary. Subsequently, an occipital lobectomy was performed. An anatomical study of the nodule confirmed the observations described in Fig. 2.

After surgery, the patient experienced a favorable postoperative course without the development of new neurological symptoms. As of the latest follow-up, hemianopsia persists, but there is no evidence of CF relapses. The patient has successfully returned to work and is able to lead a normal life.

## Discussion and conclusions

Cyst formation and CEEH after SRS treatment of AVMs are complications scarcely described in the literature, especially regarding their pathophysiology and treatment. CF is defined as the development of a fluid-filled cavity within or adjacent to the location of the original AVM nidus, despite cerebral angiographic confirmation of total AVM obliteration [2]. On the other hand, CEEH represents another rare complication, distinguished by the presence of a subacute expanding hematoma surrounded by a capsule [3, 7]. It is determined radiographically as a low-signal or mixed-signal intensity lesion on T2-weighted MRI [4]. These complications differ from early radiation-induced changes (RIC) observed within the first 1 to 2 years after AVM SRS, defined as perinidal T2-weighted hyperintensity signal on MRI, and radionecrosis [12].

The incidence of CF is generally believed to be below 5% [2, 9, 10, 13] while CEEH is considered an even rarer complication, with an estimated incidence of less than 1% [3, 6]. However, when considering both entities together, the combined incidence can increase up to 7% [8]. There is a prevailing concern that these late complications may be underestimated due to inconsistent follow-up durations across patient series and the potential for asymptomatic and self-limiting presentations [2, 9]. In our case, the onset time between SRS and CF was 10 years, consistent with findings reported in the literature [10–14].

### CF vs CEEH

The literature concerning CF and CEEH can be confusing, as they are often described as separate entities that may coexist [4, 6, 11]. For instance, Abou-Al-Shaar et al. [6] recently reported a series of 15 patients with CEEH, four of whom also had a cyst associated with the hematoma. Pomeraniec et al. [2] reported 17 cases of cyst formation, three of which experienced post-SRS hemorrhage. Therefore, the association between these conditions is not well-studied, resulting in overlapping terminology and descriptions.

To our knowledge, only two comparable cases are documented in the literature. Park et al. [11] reported a series of patients diagnosed with CEEH, one of whom had a concurrent cyst formation. Partial evacuation of the CF/CEEH resulted in recurrent bleeding and worsened symptoms, with subsequent neurological decline. However, the presence of an angiomatous nodule was not described, and the authors identified partial capsule removal as the primary reason for relapse. Similarly, Nakamizo et al. [15] described a single patient who initially underwent CT-guided cyst aspiration and later developed a CEEH requiring resection.

In our case, despite undergoing craniotomy and surgical cyst resection, a relapse of the condition occurred, manifesting what has been described as CEEH. This outcome leads to two interpretations. Firstly, contrary to the prevailing conception that CF and CEEH are distinct entities, both scenarios may, in fact, represent a spectrum of the same condition.

Secondly, our case highlights the central role of the reddish nodule in the development of both CF and CEEH. It has been hypothesized that the cyst forms primarily due to repeated hemorrhage from the angiomatous lesion into the cavity [13]. Additionally, our case lends support to the hypothesis proposed by Shuto et al. [13], suggesting that the development of CF or CEEH may depend on the relationship between the angiomatous lesion and the hemorrhage.

Similarly to previous literature, [13, 14] pathological examination of the nodule revealed increased capillaries with thickened walls and macrophage infiltration, as depicted in Fig. 3. This unexpected evolution and the characteristics of the described lesion led us to consider the possibility that the patient’s condition was a cavernous angioma secondary to SRS, which is a known and reported entity [16]. However, both radiological and anatomopathological features make this scenario less likely.

### Treatment

Furthermore, this case underscores the necessity of complete surgical removal of the angiomatous nodule. While the optimal treatment for post-SRS cysts remains undefined, initial observation and follow-up are typically recommended, as some cysts may regress spontaneously [17]. Medical therapies such as bevacizumab or dexamethasone have been proposed, yet their efficacy in post-SRS cysts lacks conclusive evidence [9]. When cysts exhibit growth, mass effect, or become symptomatic, surgical intervention becomes necessary [2, 13]. Various approaches, including Omaya reservoir or cysto-peritoneal shunt placement, have been suggested. However, the benefits of intrasurgical removal of the nodular angiomatous lesion remain unverified to date [2].

This report highlights the necessity for a reassessment of management strategies regarding cysts associated with angiomatous nodules. Initially, conservative management of the cyst was pursued with serial MRI monitoring and dexamethasone administration. However, as the cyst continued to enlarge, causing an increasing mass effect and the identification of intracystic septations, surgical intervention became imperative. Other less aggressive management strategies, such as puncture of the cyst, were considered, but we lacked confidence in the significance of the contrast-enhancing nodule, as the literature on this topic is scarce and its physiological role remains uncertain. Consequently, a craniotomy and cyst fenestration was performed; however, subsequent outcomes indicated that this approach was insufficient, likely due to incomplete removal of the angiomatous lesion. This underscores the importance of aggressive cyst resection in cases where cyst enlargement is accompanied by an associated nodule. Simple cyst or hematoma drainage procedures or Ommaya reservoir placement may prove inadequate and could lead to the recurrence of the condition, potentially increasing morbidity and mortality. Therefore, complete resection, with careful consideration of the associated nodule, should be considered as the initial therapeutic modality in cases of symptomatic CF/CEEH, considering the specific brain region involved and the anticipated morbidity.

Retrospectively analyzing our case, we can identify several factors that could have predicted a high risk of developing cystic formation as a late complication since: RIC could be found 5 years after the first SRS treatment was performed, the location of the AVM was lobar, a complete obliteration was achieved, hemorrhage occurred as the clinical presentation and SRS treatment was performed with a LINAC [2, 7, 9, 10, 12].

Consequently, serial MRI follow-up is highly recommended even years after confirming AVM occlusion by DSA. All mentioned factors should be considered when determining the frequency of follow-up MRI scans. The potential implications of CF/CEEH, affecting up to 7% of AVMs treated with SRS, justify the necessity for prolonged patient monitoring, even in cases of asymptomatic complete AVM obliteration. Special emphasis should be placed when RIC is observed.

## Conclusions

The present case highlights that despite the varied terminology used in the literature, both CF and CEEH represent different manifestations of the same underlying pathology, best understood as a continuum. The angiomatous nodule has an important role in its physiopathology. When CF/CEEH presents with symptoms or exhibits growth, aggressive intervention is warranted, particularly targeting complete resection of the angiomatous nodule.

## Data Availability

Data sharing is not applicable to this article as no datasets were generated or analyzed during the current study.
